# A qualitative study of perceptions of a mass test and treat campaign in Southern Zambia and potential barriers to effectiveness

**DOI:** 10.1186/s12936-015-0686-3

**Published:** 2015-04-21

**Authors:** Kafula Silumbe, Elizabeth Chiyende, Timothy P Finn, Michelle Desmond, Chilunga Puta, Busiku Hamainza, Mulakwa Kamuliwo, David A Larsen, Thomas P Eisele, John Miller, Adam Bennett

**Affiliations:** PATH Malaria Control and Evaluation Partnership in Africa (MACEPA), Lusaka, Zambia; Center for Applied Malaria Research and Evaluation, Tulane University School of Public Health and Tropical Medicine, New Orleans, LA USA; National Malaria Control Centre, Ministry of Health, Lusaka, Zambia; Department of Public Health, Food Studies and Nutrition, Syracuse University David B Falk College of Sport and Human Dynamics, Syracuse, NY USA; Malaria Elimination Initiative, Global Health Group, University of California, San Francisco, 550 16th St., San Francisco, CA 94158 USA

**Keywords:** Mass Test and Treat, Malaria elimination, Qualitative methods

## Abstract

**Background:**

A mass test and treat campaign (MTAT) using rapid diagnostic tests (RDTs) and artemether-lumefantrine (AL) was conducted in Southern Zambia in 2012 and 2013 to reduce the parasite reservoir and progress towards malaria elimination. Through this intervention, community health workers (CHWs) tested all household members with rapid diagnostic tests (RDTs) and provided treatment to those that tested positive.

**Methods:**

A qualitative study was undertaken to understand CHW and community perceptions regarding the MTAT campaign. A total of eight focus groups and 33 in-depth and key informant interviews were conducted with CHWs, community members and health centre staff that participated in the MTAT.

**Results:**

Interviews and focus groups with CHWs and community members revealed that increased knowledge of malaria prevention, the ability to reach people who live far from health centres, and the ability of the MTAT campaign to reduce the malaria burden were the greatest perceived benefits of the campaign. Conversely, the primary potential barriers to effectiveness included refusals to be tested, limited adherence to drug regimens, and inadequate commodity supply. Study respondents generally agreed that MTAT services were scalable outside of the study area but would require greater involvement from district and provincial medical staff.

**Conclusions:**

These findings highlight the importance of increased community sensitization as part of mass treatment campaigns for improving campaign coverage and acceptance. Further, they suggest that communication channels between the Ministry of Health, National Malaria Control Centre and Medical Stores Limited may need to be improved so as to ensure there is consistent supply and management of commodities. Continued capacity building of CHWs and health facility supervisors is critical for a more effective programme and sustained progress towards malaria elimination.

## Background

Zambia has demonstrated considerable success in scaling-up recommended malaria control interventions over the past decade and shown corresponding reductions in malaria morbidity and mortality [1]. Following these successes, the recent National Malaria Strategic Plan called for ambitious efforts to work toward malaria elimination and the establishment of at least five malaria free zones by 2015 [2]. To achieve these objectives, the Ministry of Health (MOH) in conjunction with the Malaria Control and Elimination Partnership in Africa (MACEPA) implemented a mass malaria testing and treatment (MTAT) intervention with artemether-lumefantrine (AL)(Coartem®) (MTAT-AL) in Southern Province, Zambia. The MTAT-AL intervention aimed to reduce malaria transmission through testing the entire population with a rapid diagnostic test (RDT) and treating all infected individuals, thereby targeting the parasite reservoir in the population both amongst individuals experiencing symptoms and likely to seek treatment at a facility, as well as amongst individuals not experiencing symptoms but still infected.

The effectiveness of the MTAT-AL intervention to reduce parasite prevalence and health facility incidence was based on the assumption that the majority of infected individuals would have high enough levels of parasites or antigen in their blood to be detected by the RDT at the time of screening [3]. However, previous modelling efforts have suggested that up to 50% of infections may be missed by microscopic testing and RDTs [4], and preliminary results of the MTAT campaign suggest only modest reductions in parasite prevalence and health facility incidence were achieved [5]. A similar community wide test and treat campaign in Burkina Faso found no effect on malaria morbidity [6]. As a result of these findings, new drug regimens and focal presumptive treatment approaches are under consideration for future test and treat campaigns.

A primary goal of MTAT is the reduction of parasitaemia in the population amongst individuals not experiencing symptoms severe enough to motivate them to visit a health facility. As a result, the success of these interventions depends largely upon the participation of the large majority of the population, even when the perceived personal benefit may be low [7]. However, little is known regarding individual and community perceptions, acceptability, and adherence associated with these community-wide test and treat activities and how these factors may influence programme effectiveness. In-depth understanding of these factors is, therefore, crucial to interpreting the results of completed intervention rounds, and improving the effectiveness of the programme for future rounds and developing strategies for scale-up in new communities. Previous research suggests that while general knowledge of malaria and appropriate treatment for clinical malaria may be high in target communities, understanding the treatment rationale, and, therefore, adherence with treatment, may be low for individuals not experiencing symptoms during a test and treat campaign [8]. Additionally, there may be difficulties associated with delivery of these interventions through community health workers (CHWs) if they are not seen by the community as properly trained [8].

This paper reports results of interviews and focus groups that were conducted with community members receiving the MTAT programme, CHWs conducting the screening and treatment activities, and health centre and MOH officers involved in coordinating the intervention in order to elucidate perceptions of the intervention, perceived benefits, potential challenges that may have limited effectiveness, and needs for programme sustainability.

## Methods

### Study site

The MTAT intervention was conducted in four districts—Gwembe, Sinazongwe, Siavonga, and Kalomo—in Southern Province, Zambia, which border Lake Kariba and Zimbabwe to the south and east, and Western Province to the west. The population of Gwembe, Sinazongwe, Siavonga, and Kalomo districts totaled 369,856 in 2012. Qualitative data collection was conducted only in Gwembe and Siavonga districts. Malaria transmission is highly seasonal, peaking following the rains from November through April, and focused within these districts along Lake Kariba. The mean annual parasite index (API) from 2009–2011 was 917 per 1,000 population in Gwembe, 612.5 per 1,000 in Sinazongwe, 66 per 1,000 in Siavonga, and 75.7 per 1,000 in Kalomo (National Malaria Control Center programme data).

Southern Province was selected for the MTAT intervention due to a progressively lower malaria disease burden achieved through relatively higher coverage of proven interventions over the period of scale up from 2005–2010. Results of Malaria Indicator Surveys (MIS) conducted in 2006, 2008 and 2010 showed that coverage of primary preventive interventions [insecticide-treated nets (ITNs), indoor residual spraying (IRS), and intermittent preventive treatment for pregnant women (IPTp)] increased to high levels preceding the MTAT intervention: the proportion of households with either an ITN or IRS increased from 49.1% in 2006 to 75.4% in 2008 and 75.6% in 2010 in Southern Province [9]. Furthermore, these surveys showed an overall decreasing prevalence of malaria parasitaemia (15.5% in 2006, 7.7% in 2008, and 5.5% in 2010) among children less than five years of age in Southern Province [9].

The National Malaria Control Centre has been rolling out malaria case management services (called home management of malaria) since 2006 in various parts of Zambia with largely donor funding. The scale of activities had not received full scale across Southern Province prior to the MTAT campaign, although some CHWs had participated in previous trainings. Further, as national interests shifted from home management of malaria to integrated community case management (iCCM), including the management of pneumonia and diarrhoea, CHWs were provided additional training by various partners. Benchmarking of malaria case management systems effectiveness suggests that even by 2011–2012, coverage at community levels across the country were not optimal [10]. These earlier trainings undoubtedly laid the groundwork for local acceptance of testing and treatments for MTAT, and MTAT planning efforts were careful to target more complete coverage and engagement of CHWs in the process.

### MTAT campaign

**Figure 1:**
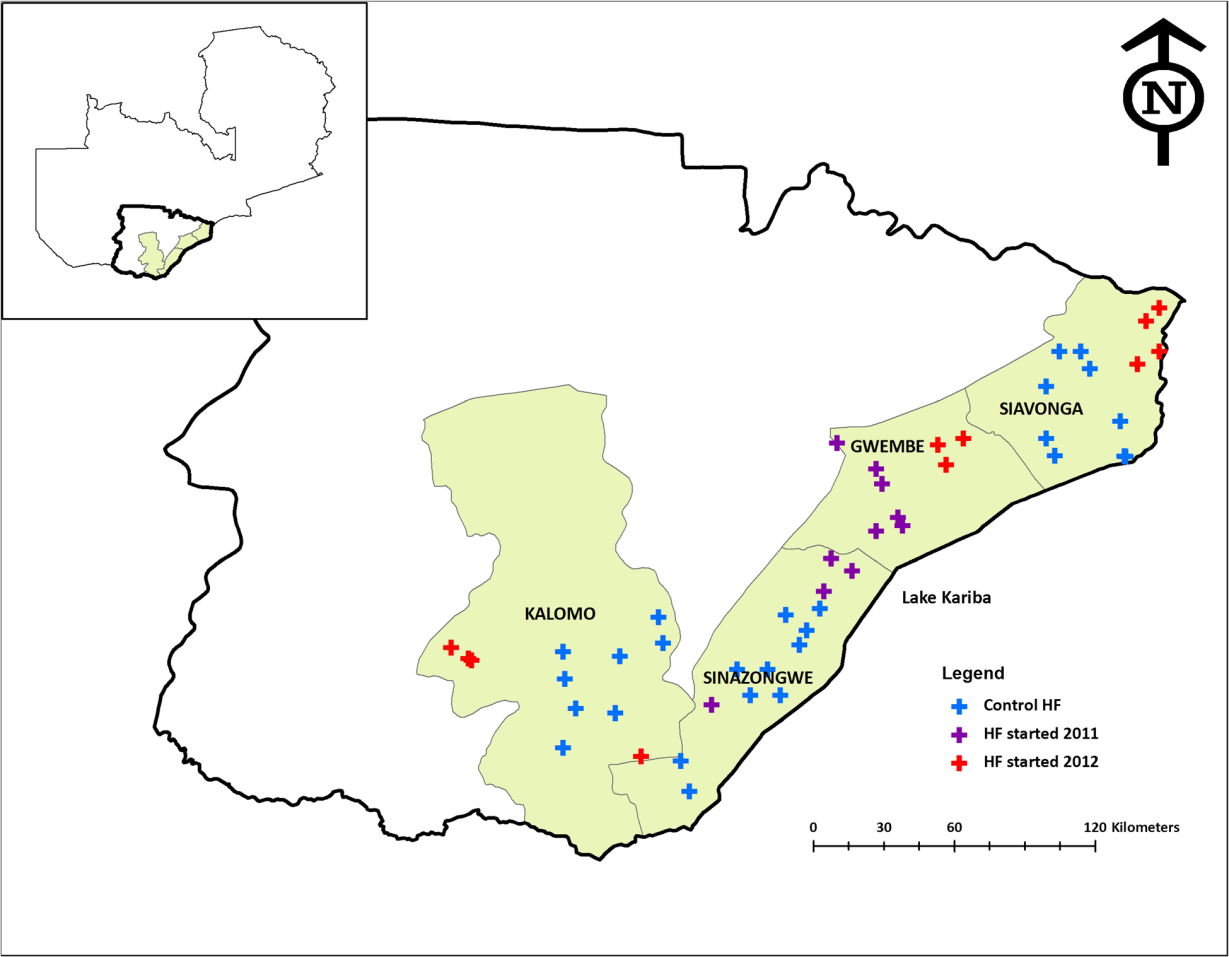
Mass Test and Treat study districts and health facilities (HF) in Southern Province.

Initial implementation of MTAT activities was piloted in selected health facility catchment areas in Gwembe and Sinazongwe districts (Figure 1) in November 2011 and January 2012, with roughly 50,000 community members tested. MTAT activities were expanded to include all four intervention districts with three rounds during the following low malaria transmission season. In 2012, MTAT rounds were conducted in all four intervention districts in May/June, July/August, and September/October, with over 80,000 individuals tested in each round. During each round, CHWs from intervention facility catchment areas conducted a complete household census of their communities and tested every household member using RDTs. All individuals testing positive were then given a full course by weight of AL.

Prior to the intervention, CHWs were trained on malaria case management, storage of used RDTs for further molecular analysis, uncomplicated malaria treatment and referral for severe malaria, and in the use of personal digital assistants (PDAs) for data collection. At the time of testing and as part of the surveillance efforts, CHWs asked if any household member had been ill with fever during the previous two weeks, if treatment was sought, and if the household owned any ITNs. In the event that any household members with recent history fever were not present during the household visit, or if any household members tested positive for malaria, the CHW scheduled a time to revisit the household to test these individuals. During implementation, ITNs were also distributed to households that notably did not possess enough ITNs to cover all the sleeping spaces or did not own an ITN at all.

### Sampling for qualitative data collection

Gwembe and Siavonga districts were selected for qualitative data collection based upon the longer duration of implementing MTAT activities (for Gwembe), and the fact that these districts reported more challenges during the early course of implementation. Within Gwembe and Siavonga districts, MTAT-implementing health facility catchment areas were randomly selected for qualitative data collection. Selected catchment areas were then purposively assigned to interview or focus group type, as described in Table 1.

**Table 1:** **MTAT Qualitative study district level interviews and focus group discussions**

**Administrative level**	**Focus group discussions**	**In-depth interviews**
Siavonga District	1 group of 12 Community Health Workers (CHW), 2 groups of 12 women each, 1 group of 12 men	5 District Medical Office (DMO) staff, 3 health facility staff, 5 CHWs
Gwembe District	1 group of 12 CHWs, 2 groups of 12 women each, 1 group of 12 men	5 DMO staff, 3 health facility staff, 5 CHWs
Southern Province		2 Provincial Medical Office staff
National Level		5 Interviews with MOH staff and Medical Stores Limited staff

Community members in selected MTAT catchment areas were asked if they would be interested in participating in focus group discussions (FGDs) to discuss the intervention. Selection of individuals from communities was conducted to maintain gender balance. Similarly, CHWs conducting the intervention in these areas were asked to participate in separate FGDs and in-depth interviews (IDIs). Finally, key informant interviews were conducted with health facility supervisors and district health staff in the selected districts, as their role was to provide technical direction and oversee logistics and the activities of the catchment teams within their district. All those eligible for interviews were informed by phone, email or word of mouth to make an appointment for the interviews to be conducted.

### Training for qualitative data collection

Qualitative data collection was conducted by the Malaria Focal Point Persons (MFPPs) for Sinazongwe and Kalomo districts and two district-level staff members from Gwembe and Siavonga districts to ensure equal district representation on the data collection teams. All four data collectors were conversant in Tonga, the local language spoken in the study areas. Two MACEPA staff members were assigned as team leaders, such that each data collection team comprised at least three individuals.

Data collection commenced immediately following a three-day training on the intervention, interview techniques, and practice and use of interview guides. Data collection was conducted over a two-week period from February 8–22, 2013. At the same time a study team member was assigned to collect data through interviews at the MOH headquarters, National Malaria Control Centre (NMCC), and Medical Stores Limited (MSL) in Lusaka.

### Interview guides

Interview guides were developed to structure both FGDs and IDIs. The interview guides included open-ended questions designed to elicit responses to the following questions regarding MTAT campaign implementation:

1. How do community members feel about repeated malaria infection screening and treatment and what are possible reasons people may refuse the intervention?
2. Are individuals testing positive complying with treatment regimens, and what are potential reasons for treatment non-adherence?
3. How is the MTAT campaign affecting community health workers?
4. Has the increased malaria surveillance data influenced the behaviors and perceptions of health workers, and if so how?
5. Can the MTAT campaign be scaled beyond the current target districts, and what would be required to do so?

### Focus group discussions

Eight FGDs were held with groups of no more than 12 community members or CHWs each. Each FGD lasted from a minimum of 90 minutes to a maximum of two hours and was conducted in the Tonga language. All FGDs were recorded and all participants consented to being recorded. The FGD facilitator followed the interview guide, and interviewees were free to ask any further questions related to the study as well as overall malaria control and prevention activities.

### In-depth interviews

A total of 33 IDIs were conducted with CHWs, provincial health level staff, and MOH, NMCC and MSL health officers in Lusaka. This was to ensure that perspectives were included from all levels of health care system involved in MTAT activities. All IDIs were recorded on a voice recorder and all interviewees consented to being recorded. Once the interview was concluded the recorder was stopped and interviewees were free to ask any further questions related to the study and thanked for their participation.

### Transcription and data analysis

At the end of the data collection exercise, all voice recordings were translated from Tonga to English and transcribed into text for analysis. Data analysis followed a Grounded Theory approach, whereby a codebook was developed based on the themes defined by the interview guide and initial reviews of interviews and focus group transcripts, and updated in an iterative fashion to form similar conceptual categories [11]. Coding of the data was conducted using the software program Atlas.ti v.7 [12]. The primary coder began by reviewing all transcripts and assigned codes to portions of interview text based upon the agreed upon code definitions developed by the team. A second coder reviewed the transcripts for consistency and the analysis team adapted the codebook according to preliminary findings, and guided by the original research questions.

## Results

The results of FGDs and IDIs are organized under subheadings corresponding to the research questions and themes derived from the coding process. These themes include: community and CHW perceptions of the MTAT campaign; challenges experienced by CHWs and district staff; refusals and non-adherence in the community; and needs for programme improvement, sustainability, and scale-up.

### Community perceptions of the MTAT campaign

Focus group discussions with the MTAT community members indicated that in general they were pleased with the MTAT programme. As expressed by a community member: *“People have started accepting to be tested and if found with the parasite they were treated there and then”.* Another emphatically stated: *“This programme should continue because nowadays we feel that our health is good in terms of malaria disease. I am happy because all the time I am able to see, if at all I have malaria or not, so I am really happy because all the time my children are able to know if there is any malaria and to trust that they are doing fine in terms of being sick of malaria”.*

Community members appreciated the programme because they perceived it as being good for the health of their families as well as having the capacity to reduce malaria, as the following quote from a community member illustrates:

“*I am thankful because malaria has reduced, there used to be so much malaria especially when maize is about to be eaten. Here [*at the clinic*] there are no people on these benches-- before there used to be long lines under those trees, on these benches, and the veranda used to be filled with malaria people, so right now malaria has reduced. So I am encouraging that this work should not stop but continue, maybe this disease can leave us”.*

An additional stated benefit of the programme from the perspective of the community was not having to walk long distances to the health facilities to receive malaria services, as one community member stated: “*people are happy because they are being visited in their homes. Even if you did not prepare to go to the hospital, they come and treat the malaria you have. So people are really happy in the village because of the same”.* Another community member observed:

“*I would encourage them to continue with the programme of testing us from our villages because if they stop we are going to suffer a lot. You find that an elderly person is sick [and] no one is able to bring him/her to the hospital, so if they come and do the testing [that is better] because there is no one to take them to the hospital”.*

Combining MTAT activities with ITN distribution evoked mixed responses from the community. One community member expressed the following:

*“Me, I am just happy that they help us to prevent this disease through this programme…instead of walking all the way here, you find that they bring you medicine and distribute mosquito nets so that we prevent malaria, so we are just encouraging them to see us when they come again that is through this programme. We thank them in that they protect us through mosquito nets and treatment of malaria*”.

However, another community member expressed some concerns with the ITN provisions associated with the campaign: “*The only thing I will complain about in this malaria programme is that they protect us but they only give one mosquito net. In my household I have a son who is 7 years old, a small child, and also I have a school going big boy, let me just say there are three beds, then they just gave me one net where my husband and I sleep under, the other two there is nothing. So because of not wanting the children to die– instead it is better we the adult die– I gave my net out to the children so that I can sleep without, that is all I ask for so that they can include some more so that we all benefit”.*

### Community health worker perceptions of the MTAT campaign

Key informant interviews and FGDs demonstrated that in general, CHWs involved in conducting test and treat activities understood the rationale for the MTAT intervention, as illustrated by a quote from one health worker: *“In my opinion [MTAT] is one of the interventions where we want to target the communities in order to further reduce malaria in terms of transmission and eventually reduce the burden, where health workers go into the communities and screen the members of the community using a Rapid Diagnostic Test, and those found to be positive are treated, and this also helps us to identify places where there are hot spots of malaria, where there is focal transmission…..For me it’s an extra intervention from the usual interventions to do with prevention in malaria”.*

Similar to community member responses, there was a great amount of satisfaction expressed by CHWs involved in programme activities. Some of the key themes that emerged around satisfaction with the programme included a perceived reduction in the malaria burden, the ability to reach people who live far from health services, and acquisition of knowledge and new skills. In the words of one CHW:

“*I am happy because from the time we starting the malaria testing in this village, malaria in the villages has reduced, nowadays you can go round the villages, it is not like it used to be at all.*”

Several CHWs indicated satisfaction in their ability to reach people who live far from health services, who might otherwise have to travel long distances. In the words of one CHW: *“it makes me happy because the distance from the villages to the health clinic is far, so I help the people from those villages by carrying medicine for them and treating them in their villages*”.

A further commonly reported positive aspect of MTAT by CHWs was the acquisition of new knowledge and skills, including greater knowledge of malaria. In the words of one CHW: *“I have learnt a lot because I did not know what brings malaria is mosquitoes, I thought maybe what brought malaria is sugar, but through being sensitized know that what brings malaria is mosquitoes”.*

Additionally, some reported that the learning process extended to issues beyond malaria, as another CHW shared: *“We learned that when we reach the village we must respect the people, not forcing them, talking to them with respect, greeting them very well. We were taught very much; if a person insults you don’t return”.*

### Challenges experienced by community health workers

In addition to these positive experiences, CHWs reported numerous challenges in conducting the campaign. The most common themes related to challenges encountered during implementation of MTAT included inadequate transport, the need to cover long distances, problems with PDAs, and inadequate compensation and supplies.

Numerous CHWs complained that transport provided for them to conduct MTAT activities was insufficient. Often the allocated vehicles were so few that they were required to make multiple trips to intervention sites, or to share vehicles between several catchment areas. As one CHW lamented: *“transport is not working well for us; Sinafala, Chipepo Secondary and Chabbobboma all depend on one source of transport, so when it does not show up we start off because we work with time, so we carry our luggage to go … the vehicle will meet us with our luggage”.*

Several CHWs reiterated that although the MTAT benefited community members by allowing them to receive health care services at home, CHWs were required to walk long distances in order to provide these services. CHWs were required at times to cover distances of up to 20 km on foot due to lack of transport. In the words of one CHW: ***“****we don’t have transport to use, [and] we walk long distances, so we felt it could be better if each centre could have a vehicle to use because we work from 3 centres, at least if they would get us and drop us some where instead of [us] walking long distances”.*

PDAs were used to record testing and household data during MTAT campaigns. However, the testing teams encountered challenges in keeping PDAs charged as most live in houses without electricity, and although some had access to solar chargers at health facilities, these did not provide enough chargers for all the PDAs. In some cases this disrupted or delayed testing activities. As one CHW observed: *“we also find problems in the charging system, like the PDAs that we use, if the batteries are flat then the work will not move well at all…. we don’t have solar to charge with and we don’t find electricity where we go…”.*

Another CHW noted:

“*So charging of the PDA was a big challenge and even it made our work to be delayed because you have to go and ask from those people who have panels [and] pay them a bit of money, buy fuel and pay them so that they can charge your PDAs. Like these people who have solar panels they would maybe charge them early in the morning maybe up to 10 hrs and people would start work late, maybe after 10 hrs to 12 hrs”.*

As the MTAT programme was implemented through CHWs who are volunteers, CHWs and facility staff felt that appropriate incentives to maintain motivation would be essential for sustainability and programme scale-up. Study participants observed that incentives such as lunch allowances, transport and uniforms were a great motivation for the CHWs.

Another key theme shared regarding challenges faced by CHWs during the MTAT campaign programme was inadequate supplies. Coartem (AL) and RDTs were supplied continuously based on requests made by each district from MSL using the national drug supply and logistics routine distribution mechanism. Other supplies such as sharp boxes, gloves and swabs were supplied by NMCC and MACEPA during programme implementation; these were distributed to the district health office, health facilities, and finally health facility catchment teams. However, in the early months of implementation, some teams experienced stock-outs of AL and RDTs, which was attributed to inconsistent coordination between the MOH and MSL. While this situation had improved at the time of the interviews, facility health staff and CHWs felt that it would be essential to ensure that programme requisites are consistently available. A CHW explained: *“I remember the first round I think we ran out of commodities so we had to produce [look for] extra rapid diagnosis tests”.*

### Reasons for refusing to participate

Participants in the community FGDs acknowledged that some members of the community did not readily participate in the MTAT activities. The primary reported reasons for refusing to be tested included suspicion that CHWs could be practicing Satanism and may use their blood for rituals, fear of collected blood being sold or used to test for HIV infection, other uncertainties about how the collected blood would be used, and anxiety about the entire process of testing and treating. Personal religious beliefs and not feeling adequately informed about the study also contributed to refusals to participate.

One community member stated the following as a reason for refusing to be tested by CHWs:

*“Others think it is Satanism. People know that getting blood is associated with Satanism. People think that blood is going to be sold somewhere. So people had such problems”.*

Others observed that refusals may occur among some community members belonging to some churches which insist that their people should not test or drink conventional medicine because healing comes from God. In the words of a community member:

*“In our area there is one from a certain church who refuses because they worship their God who sustains them”*

The fear that their blood would be used to test for HIV infection was a common reason for refusals, as one CHW noted:

*“Others refuse because they think you want to test them for HIV and AIDS and others think the blood I take maybe I want to take it somewhere or buy vehicles because of their blood”.* Another CHW iterated “*the reason they refuse other than the church is because of the test; in these villages people don’t know to read even when the t-shirts are written they think we are there to test HIV/AIDS*”.

In addition to these cultural and literacy barriers, participants described inadequate information as another reason why communities refused to participate in MTAT activities. The lack of information ranged from people not knowing enough about the intervention and the inability to dispel some of the existing myths around testing.

Some community members refused to participate in MTAT because they believed they could not have malaria, as they did not feel sick, as one CHW shared:

“*Actually that was where we experienced some difficulties; it’s like people could not understand the importance of being tested… what we told them was that once they are tested we were going to find that malaria was going to be there because it could be just that people are carriers… So we educated them in all that, saying if you are a carrier it doesn’t matter, but if you are having that infection you are going to keep on infecting other people so mosquitoes which bite you will just be getting an infection from you and giving it to other people. So it’s better that each one of us is tested so that we can remove that malaria parasite from our bodies and our communities where we are staying. And they accepted that though it was a little bit hard for these people…”*

A minority reported that refusals could result from incentives paid to the CHWs that were conducting the test and treat campaigns. One CHW highlighted this issue in the following words:

*“Others were saying we don’t want to be tested because our friends who are testing are given money (sorry for me to say such a thing), but with us who are being tested, why don’t they give us maybe K5.00 ($1) or whatever thing. So we should also be given something for testing our blood”.*

Additionally it was reported that some of the community members refused to be tested for malaria because they felt that CHWs did not have adequate skills and knowledge to conduct the activities even though they had undergone training. As one community member said**:**

**“***I think people were a bit suspicious now, how can this one hammer me an injection and yesterday we were together in the village. Which of course- you know even for teaching I may not just come from the village and come in class and start teaching, no!”*

Respondents further disclosed that there was a need to provide more training to equip the CHWs to do this work efficiently. In the words of a community member: *“We are happy that they employed the (indigenous) people but let them take them to a special training where they can learn how to handle these things because some of them could even fail to prick people - those could be some of the challenges that made people to shun away from the activity”.*

### Reasons for non-adherence with treatment regimens

An important aspect of this study was to ascertain the extent to which those treated for malaria actually adhered to the prescribed drug regimen. The general consensus among the community and CHWs was that people on treatment largely adhered to the drug regimens; however, respondents agreed that some did not adhere due to reasons including inadequate information given to the person on the rationale for treatment, lack of understanding of the benefits of completing the full course for malaria treatment, religious beliefs, and simply not wanting to take more drugs once they felt better. These are reflected in a statement by a community member:

“*Some they continue, others not. They only take the first and second then they stop saying they are healed. They also give their children when they are not well in their bodies disrupting their own course. When any member of your family is sick don’t give him your medicine, take them to the clinic as well so that they can receive their own medication. So others they follow while others they don’t, they just take first, second and third stop”.*

Another community member noted “*you will find a sick person takes medication in the morning and feels well then he stops taking medicine there and then. They hide the remaining medicine so they can use the other time. That element is there in people*”

Yet another CHW observed: “*Some are not difficult, they finish, but others who don’t know the goodness don’t take, but a lot appreciate because they know that they would prevent the disease before falling sick”*

The perception of not feeling sick was a common hindrance to completing drug regimens: “*The difference is you will find that others are not sick or complaining but will be found positive, so when we give him/her medicine the difference is he/she won’t put her mind to the medicine…most of the time some don’t finish and that is the difference. So those that come and are tested when they are given medicine, they drink because they are feeling sick or are complaining….*”

Religious beliefs were also reported to cause non-adherence to treatment regimens, as one CHW shared: *“Like saving the drug it happens that I will drink when I fall sick but mostly we just find challenges with people who go to a certain church who are not using drugs, but their number has started reducing. Others have started taking the drug*”.

Social habits were also cited as a cause for non-adherence as stated by one CHW: “*What caused others not to finish their medicine is beer. When they go for beer drinking they forget their medical course, they drink in the morning, but in the evening he is drunk, he comes home from the tavern at 24 hrs he will not take his medication, he will just go straight to sleep. He can’t finish his medication”.*

### Needs for programme improvement, sustainability, and scale-up

National, provincial, district and health facility staff were asked to address scalability, sustainability, and suggestions for MTAT improvements. As highlighted by previous responses, the MTAT campaign was generally perceived as a beneficial programme that could be improved and possibly scaled-up for more effective results. Further discussions with interviewees revealed that sustainability and scale-up of MTAT would rely on a number of issues being addressed including: greater capacity-building of CHWs and MOH personnel, increased sensitization of the target populations, improved coordination of supplies and logistics between central and community levels, and improved communication at all levels of the campaign.

Several health workers commented on the need to conduct more training for CHWs to enable them to carry out the MTAT duties effectively. One CHW commented:

*“And then you need to train people, health workers including community health workers and must understand the importance of this intervention especially of going to the community and treat and screen and test and then treat within the community”.*

Another interviewee noted that in as much as it was appreciated that local people were being empowered with skills and knowledge during the trainings, it would be good to increase the duration of the training for CHWs to increase their competency: *“[There] should be ample time for them to be doing the training. We are happy that they are from the village. Why not train them for a long time so that they keep on sustaining these programmes in the village”.*

Participants reported that in order to improve acceptance of the programme and reduce refusals, it is imperative to sensitize communities on the MTAT programme. As a CHW observed: *“One of the things that needs to be improved on is the sensitization part, it has not been done so effectively and I think people need to go flat out in the field to go and do more sensitization because people don’t understand the programme*”.

Another emphasized the need to utilize community leaders for this purpose “*If we sensitize the local leaders and they understand the programme [and] they accept it, then we can get the local leaders the chiefs, headmen, political leaders. We can actually give them a message, have it recorded and have it broadcasted either on the radio, television or public address system, because we go round and inform them using the recorded message. Listening to the voices of their own leaders can increase the acceptance levels in the community because they are getting voices of their own leaders. I think if we used the local leader in every corner of our area I think we have may be 100% acceptance”.*

A key theme that emerged regarding sustainability of the programme was the need to improve supply and logistics coordination. Interviewees mentioned that the teams experienced stock outs of AL, RDTs, and ITNs/LLINs and that in the future it would be necessary to ensure stocks are continuously available for the MTAT programme to succeed. In the words of an interviewee from the national level:

*“The only problem probably which we incurred was the supplies. Probably before we started the programme we did not prepare adequately and then we relied much on Medical Stores. In the midst of the programme we incurred a lot of problems, like the gloves we had to borrow from other Districts, and probably the Coartem was okay we had enough supplies but what was difficult was the RDTs at one time in the country, the Medicals Stores didn’t have”.*

Although only mentioned in interviews at provincial level, it was evident that while communication channels were clear between the district and provinces, there was inadequate communication to facilitate staff from the provincial office to provide technical support to the districts, as noted by an interviewee from the provincial level:

*“I think we are doing well apart from the communication at provincial level and maybe getting the staff at the provincial health office a little bit more involved because you need to understand that our mandate as provincial medical officers is giving that technical support and technical backstop to the districts.*

## Discussion

In this study, qualitative methods were used to assess community perceptions, acceptability, and adherence associated with the MTAT intervention in four districts in Southern Province, Zambia. In general, participating CHWs and community members expressed positive perceptions about the intervention, most notably with regard to their increased knowledge of malaria and the perception that community-level testing and treating was leading to a reduction in the malaria burden. Additionally, community members held positive perceptions about receiving testing and treatment for malaria in their homes, which alleviated the need to travel long distances for care.

However, the MTAT intervention was not without challenges inherent to large-scale population-based health interventions. The long distances CHWs needed to travel to cover their areas may have affected intervention coverage. Several responded that this was a primary challenge, and that transportation to hard-to-reach areas was imperfect. Similarly, data collection with PDAs was challenging, as it required close proximity to electricity sources for recharging, which were often not available to CHWs. Finally, commodity shortages may have limited the effectiveness of field teams to reach all of their catchment areas in a timely fashion.

At the community level, misunderstandings about the rationale for the intervention due to previous interventions and cultural norms were the primary factors limiting acceptability and adherence, which may have contributed to lower community-level intervention coverage. Refusing to be tested was most commonly attributed to fear of misuse of blood samples, religious concerns and mistrust that the blood would be tested for HIV infection. Similar challenges with taking blood have been noted previously in both health facility and community settings. Comoé and colleagues reported that perceiving blood as a sacred body fluid influenced refusing an RDT test in a clinical setting, as did the concurrent use of RDT tests for HIV infection [13]. Boahen and colleagues reported fear of use of blood for rituals as a possible cultural barrier to blood draws [14]; and Nchito and colleagues reported high loss to follow-up in a longitudinal study in Lusaka, Zambia due to fears the blood would be used for ‘Satanism’ [15], which was also referred to by community respondents in our study.

Additionally, some community members stated that they did not believe other members of their community had adequate training or expertise to take blood samples and administer treatment. Properly trained CHWs in Zambia have been shown to be effective at administering RDTs and adhering to test results [16,17], and a review of community case management by CHWs found high adherence to RDT results across numerous studies [18] However, formal certification may enhance CHW status in the community and allow CHWs to more effectively conduct testing and treating campaigns with confidence from community members [8,19]. Finally, although CHWs were trained to refer severely ill patients to the nearest health facility, we did not focus on adherence to referral in this study; this has been identified elsewhere as an area requiring further study [18].

Adherence with treatment regimens may have been further hampered by the perception that continued treatment was not necessary once symptoms abated. This finding, similar to that reported by Okello and colleagues, during a school-based screening and treatment intervention in Kenya [8], and by Lemma and colleagues during routine health service delivery [20], further highlights the importance of educational campaigns conducted before and during the intervention. Where possible, use of directly observed therapy to ensure asymptomatic individuals complete treatment, may improve adherence. Studies evaluating adherence to MDA campaigns suggest that individual adherence will depend upon perceived risk of side effects as compared to the personal benefit of treatment [21,22].

To increase intervention coverage, these issues will require greater community sensitization, possibly through the use of village chiefs, to increase knowledge and awareness of intervention activities. Greater sensitization has been shown previously to directly influence participation in mass drug administration (MDA) for malaria and neglected tropical diseases and other large community health campaigns [7,22]. As reported for school-based interventions, maintaining ongoing dialogue with communities during mass campaigns is critical for dynamically addressing barriers to successful implementation [23]. A recent study on community barriers to MDA for malaria in the Gambia similarly highlighted the need for frequent communication, education, and sensitization events throughout the campaign to reduce the fear of side effects and improve understanding of taking the drug even if one does not feel sick [24].

## Conclusion

These findings indicate that the MTAT campaign was highly acceptable and was perceived by most respondents as a valuable programme for reducing the malaria burden. According to both community health workers and community members, the MTAT campaign increased access to care and reached those who would normally not be reached through the health delivery system. However, refusals and poor adherence to treatment regimens may have adversely influenced intervention coverage, and ultimately effectiveness. Suggestions for future MTAT campaigns include strengthening of community engagement through regular meetings with community leaders, and specific campaign messages created and shared through radio spots and text messages with cell network service providers.

Maintaining adequate and consistent commodity supply chains is equally essential for MTAT campaigns to achieve the desired impact. Strengthening communication activities within the various communities, at all facility, district, and provincial levels will encourage treatment adherence as well as reduce refusal rates. Furthermore, effective regular and up to date communication is needed to address knowledge gaps and ensure continuous capacity building through the ongoing interactions for CHWs and health facility staff. Central and Provincial level staff overseeing all health activities within the districts should be engaged early and often in the planning process to ensure effective management of the programme.
